# Getting Published in Medical Education: Overcoming Barriers to Scholarly Production

**DOI:** 10.5811/westjem.2017.11.35253

**Published:** 2017-12-22

**Authors:** Michael Gottlieb, Erin Dehon, Jaime Jordan, Suzanne Bentley, Megan L. Ranney, Sangil Lee, Sorabh Khandelwal, Sally A. Santen

**Affiliations:** * Rush University Medical Center, Department of Emergency Medicine, Chicago, Illinois; † University of Mississippi Medical Center, Department of Emergency Medicine, Jackson, Mississippi; ‡ Harbor-UCLA Medical Center, Department of Emergency Medicine, Torrance, California; § Icahn School of Medicine at Mount Sinai, Department of Emergency Medicine, New York, New York; || Alpert Medical School, Brown University, Department of Emergency Medicine, Providence, Rhode Island; # University of Iowa Carver College of Medicine, Department of Emergency Medicine, Iowa City, Iowa; ** The Ohio State University, Department of Emergency Medicine, Columbus, Ohio; †† University of Michigan Medical School, Department of Emergency Medicine, Ann Arbor, Michigan

## INTRODUCTION

Medical education is experiencing rapid growth with an increasing number of publications and journals dedicated to education research.^1^,^2^ Several new journals and special education issues (including the CDEM/CORD supplement by the *Western Journal of Emergency Medicine*) have arisen in recent years to address this increasing interest. As clinician educators, it is important to produce and disseminate research both for promotion and development of a subject niche, as well as to disseminate findings for others to learn from novel and successful educational interventions.

However, the quality of existing medical education research has been variable.^3^,^4^ Studies have suggested this may be due to limited mentorship,^5^ as well as challenges including available time, funding, small sample sizes, ability to navigate the institutional review board process, and difficulty with defining relevant and measurable outcomes.^6^,^7^ This article discusses five common challenges to education scholarship and provides suggestions for overcoming them.

## COMMON BARRIERS

### 1. Lack of Clarity in the Research Question

The first challenge is developing the research question. While this may seem like a relatively straightforward task, developing a* clear and important* research question evolves from an iterative process. This process generally begins with an educator’s interest in a topic and a broad research question. For example, consider the case of a program director who is interested in identifying factors related to resident burnout. This is a broad topic because the “factors” are not clearly defined nor is the hypothesis clarified. Nonetheless, this is enough information to conduct a literature review to begin to understand prior work in the area, identify where there is a gap in knowledge, identify the novel research question, and to provide a link between the research idea and a conceptual framework.

The conceptual framework is a vital component to developing a good research question, yet it is often overlooked in medical education studies. A review of published medical education studies found that 55% did not mention a conceptual framework.^4^ The conceptual framework serves as the foundation of the study that informs all aspects of the research design and should not be ignored. Frameworks relevant to medical education may be found in fields outside of medicine, especially education and psychology; so medical education scholars may want to extend their literature search outside of their medical specialty. In the example above, a thorough search of the literature would reveal that burnout studies are often framed within the context of the Multidimensional Theory of Burnout, a theory found primarily within the psychology literature.^8^ Further discussion of conceptual frameworks and how they can be used to develop medical education research projects can be found in the classic paper by Bordage.^9^

**Figure 1:**
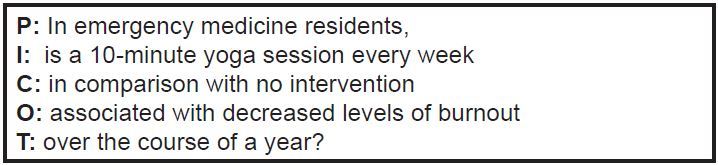
Example of a research question in PICOT formatting. *PICOT*, population, intervention, comparison, outcomes, time frame.

After conducting a thorough literature review and identifying a specific problem to address, medical educational researchers can use two mnemonic tools to further develop their research question. The first is the PICOT framework, which is used to transform a broad question into a specific one that includes all of the necessary components: **P**opulation, **I**ntervention, **C**omparison, **O**utcomes, and **T**ime frame. One study demonstrated that research reports that used the PICOT format were rated as having better overall quality than those that did not.^10^ Figure 1 includes an example of a structured question using the PICOT format (Figure 1).

**Figure 2:**
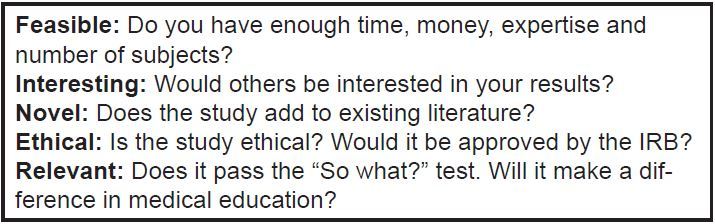
FINER criteria for assessing study questions. *FINER*, feasible, interesting, novel, ethical, relevant; *IRB,* institutional review board.

The other valuable tool for designing a research question is the FINER criteria (Figure 2). As opposed to the PICOT framework, which helps to determine that all elements of a study question are present, the FINER criteria assess the quality and likelihood of success of a given research study. The authors recommend applying both sets of criteria to a given study question to ensure that the question is adequately refined, so as to maximize the success of each academic endeavor.

### 2. Inadequate Methodology to Assess the Study Question

Editors and reviewers desire to publish interpretations that are generalizable and accurate (i.e., supported by validity evidence). Despite recommendations that medical education research adhere to the same rigorous methodological standards as clinical research, medical education research often fails in this regard.^11^ One study of submissions to a major medical education journal found that the top reasons for rejection included inappropriate statistics, over-interpretation of the results, an inadequate research instrument, an insufficient problem statement, inadequate literature review and an insufficient data, while a sound problem statement and study design significantly increased the likelihood of publication.^12^

Moreover, medical education research also fails to report substantial validity evidence, which presents a challenge to publication.^13^,^14^ Many of these threats can be minimized by choosing an appropriate study design, standardizing study conditions, and collecting and reporting detailed information about study participants and procedures.

While the measurement instruments in clinical research are typically well validated (e.g., d-dimer, troponin), education research instruments are rarely as fortunate. Therefore, not only do education researchers need to define and collect meaningful outcomes for research, they also need to ensure the validity and reliability of their measurements. Many education studies focus on novel curricula, innovations, learner behaviors, or the exploration of education concepts or environments, for which previously established instruments are unavailable. If a new instrument needs to be created, or if using an instrument from another field (e.g., psychology, sociology, secondary education), the researcher is advised to first assess the validity of the instrument with respect to the intended measurement. In order to gather enough validity evidence to support the instrument, it is essential that the instrument be matched to the goals and objectives, piloted to ensure that it performs as expected, and compared to other similar measurements or available data.^15^ The mere act of gathering validity evidence for an instrument or measurement (i.e., showing that it measures what it states it is going to measure and that it accurately distinguishes the target outcome from other outcomes) can be an important research study.

Novice education researchers faced with multiple competing demands may attempt to capitalize upon existing work by converting an ongoing education project into a research study. For example, an educator may develop a new curriculum and then subsequently decide to assess it after it has been ongoing for several months. Unfortunately, these research attempts are often unsuccessful due to insufficient planning and inadequate methodology and outcomes. To have a methodologically sound and successful study it is vital to define appropriate outcome measures at the onset and select an appropriate study design that best allows the researcher to measure the desired outcomes with minimal threats to validity. The researcher should collect validity evidence to create the assessment instrument during the developmental phase to ensure that the instrument is appropriate for the study. Involving a statistician or experienced education researcher early in the process is extremely beneficial to help avoid fatal flaws and wasted effort.

### 3. Losing Momentum

While manuscript publication should be one of the ultimate goals, it is important to set stepwise, attainable, intermediate milestones and celebrate their accomplishment on the route to manuscript completion and publication. Examples of early milestones in the scholarly process include abstract submission, initial paper development, and local and national presentations. There are also several digital mediums to publish medical education innovations. One example is the Academic Life in Emergency Medicine IDEA (Ideas in Didactics and Educational Activities) series (https://www.aliem.com/category/non-clinical/idea-series/), which allows authors to showcase novel education interventions to the broader medical education community. Additionally, educators may publish curricula online in MedEdPORTAL or the *Journal of Education & Teaching – Emergency Medicine*. Moreover, many institutions host “work in progress” sessions to highlight and discuss ongoing studies.

Ensuring early and attainable wins helps to maintain momentum for projects.^16^ To be successful, researchers must actively plan and establish short-term goals and recognize the accomplishment of these goals and the specific team member contributions throughout the process.^16^ Additionally, without a clear outline, unified writing plan, and identification of clear short-term goals, efforts can easily lose all momentum and dissolve into a disorganized, inactive to-do list without an end product. It is valuable to have regularly scheduled meetings or conference calls to ensure that all members are on track, especially for multi-institutional projects.

Education researchers should also consider maximizing the return on a given project by considering additional opportunities for expansion of a given project.^17^ Often, it requires little additional effort to convert one project into several deliverables, such as an abstract, manuscript, presentation, and digital description of the innovation. Tables 1 and 2 provide a list of potential arenas for publication, as well as examples of different formats. For example, the start of a project might include the publication of a review article or perspective on that topic. This could also be converted into a didactic for residents or training session for faculty development. As the work progresses, you might consider a reflection or short thought piece. This approach can also help maintain momentum by assisting with the early wins described above. We would like to emphasize that researchers must be conscious to avoid self-plagiarizing or artificially separating out study components to create multiple publications from a study addressing a single concept (i.e., “salami slicing”).^18^

### 4. Lack of Follow Through

Once the study is completed, it is important to go beyond the abstract with the goal of publishing it in a peer-reviewed journal. Historically, only 25–50% of abstracts presented at emergency medicine (EM) and medical education conferences are subsequently published as manuscripts.^19^–^24^ Peer-reviewed publication is important because it increases dissemination of information and is a significant consideration in achieving promotion, tenure, and future grant funding.^25^,^26^ This is particularly important for medical education research because of the relatively smaller proportion of outcomes-based studies in this field compared with clinical research.^3^,^4^

To make this process easier, the authors recommend that the researcher begin manuscript preparation at the start of protocol development, filling in components as the project progresses. Often, the introduction, research hypothesis, and methods can be drafted before the study begins, as part of the institutional review board proposal. This early planning will make the remainder of the paper more manageable when the study is completed.

When working as a team there may be more accountability to complete the paper; but team authorship can also create conflicts in author order. We therefore recommend discussing criteria for authorship, and drafting a potential order-of-authorship list, prior to beginning a study. Authors should also be aware that most publications are not accepted on the first submission, and often they may require submission to multiple journals.^27^ Authors should not let a paper linger after the first rejection. Rather, they should read the review, make appropriate edits, and quickly re-submit to another journal. Authors should also be aware that different medical education journals have different foci, and publication will be more successful if they select journals that publish similar topics or types of articles in line with their particular manuscript.

It is not unusual for authors to hit a roadblock during the writing process. This can occur at any point throughout the process from beginning to end. It can be helpful to set specific goals prior to initiating the writing process. Each goal should have a specific deadline, which can help maintain momentum and accountability. Education researchers often have many competing demands; scheduling specific times on one’s calendar for writing, similar to other appointments, can ensure dedicated time away from distractions for the author to concentrate on writing the manuscript.^28^,^29^ In order to focus on the manuscript itself, authors should avoid checking emails and other distractions.^30^ It may also be valuable to include small breaks when the author feels his or her attention waning.

Another tip is to start small. Rather than attempt to draft everything at one time, which may seem overwhelming, authors should start with either the first paragraph or the methods section (which are typically easier to write) and then expand from there. Use the standard framework for the manuscript, incorporating journal-specific components as needed. Since most articles follow a general layout, it is much easier to fill in the paper piece-by-piece using the layout as a recipe than attempting to create one’s own format.^30^ Write the first draft spontaneously and uncritically allowing for editing after the draft is written.^30^ Attempting to edit while writing can interrupt concentration and flow. Finally, authors should have other people review their work whenever possible. This provides an external perspective and helps identify errors and confusing concepts that the investigators may have overlooked. It may be valuable to include non-physician researchers (e.g., PhD, EdD, PsychD) who can provide a highly valuable external interpretation, as many reviewers for medical education journals may not be physicians.

### 5. Lack of Expertise

For faculty who lack local experts with an education research background, getting started can be difficult. In these situations, it may be helpful to reach out to the clinical researchers within or outside of the department. Likewise, many academic systems have medical education researchers in other departments or in the school of medicine who may provide guidance. Another option is to join a project team from another institution. EM research is improved by multi-site collaboration, and working with a broader group may help develop skills.

There are formal options for research skill development. These include advanced degrees such as a Masters of Health Professions Education, institutional faculty development programs in education research, or the MERC (Medical Education Research Certificate) through the Association of American Medical Colleges. Often, the EM national meetings host workshops on research methodology. In addition, finding a virtual mentor in EM or another specialty might provide support for the educational scholarship one hopes to accomplish.

## FUTURE DIRECTIONS

As medical education-focused researchers, we urge our specialty to consider future directions for creation and dissemination of our work. Just as we actively advocate for increased production, training and funding of EM clinical research, we must do the same for medical education research. First, we must start by growing the body of rigorously-conducted medical education trials published in high-quality journals. Second, we must take on the critical task of growing and promoting junior faculty who can expand our methodologic and content expertise. This step involves developing and promoting high-quality fellowships, which must include specific research training. We must encourage our mentees and colleagues to use state-of-the-art methods. This step may also consist of honest inquiry into continued barriers to methodologically-sound research studies.

Finally, to truly change the trajectory of medical education we must pursue outside funding. The sources of funding for medical education research are currently limited. As a specialty, we actively encourage government and foundation funding sources to dedicate resources to EM-relevant clinical questions. Similarly, building upon our existing contributions to medical education within and outside of EM, we must push for external groups to fund high-quality, multi-center studies of innovative educational methods. This direction should include the following: partnerships with researchers who are not primarily education focused; training of our colleagues in ways to bridge the funding divide; and possibly creation of novel funding sources (such as the Society for Academic Emergency Medicine’s medical education research grants).

## CONCLUSION

Medical education research continues to grow within EM and it is imperative that educators produce and disseminate high-quality publications to continue to advance this field. This article discusses several challenges and strategies for overcoming barriers to publication, in order to assist the educator with producing quality education research. It is the hope of the authors that this will encourage educators to publish more research to disseminate findings with the ultimate goal of further improving education and patient care.

## Figures and Tables

**Table 1:** Publication venues for medical education scholarship.

Academic Emergency Medicine Education & Training (original contributions, brief contributions, new ideas in B-E-D-side teaching (educational case reports), education case conference, commentary and perspectives, book and media review, canvas/transitions) Academic Medicine (brief report, innovation, research report, perspective, letter to the editor, last page) Advances in Health Professions Education (original research, scoping reviews, systematic reviews) American Journal of Emergency Medicine (original research, reports, correspondence) Annals of Emergency Medicine (original and brief research, literature review, commentary) BMC Emergency Medicine (original research, technical advance article, debate) Canadian Journal of Emergency Medicine (original research, review articles, updates, editorials) Clinical Teacher (original articles, insights, letters to the editor, in brief, the clinical teacher’s toolbox, faculty development reviews) Emergency Medicine Australasia (original articles, reviews, perspectives) Emergency Medicine Journal (original articles, short reports, reviews, best BETs, commentary, the view from here, swing shift: innovations in emergency medicine) European Journal of Emergency Medicine (research paper, short paper, opinion, editorial, rapid communication, review article) European Journal of Trauma and Emergency Surgery (original articles, reviews, letters to the editors) Internal and Emergency Medicine (debates, points of view, commentaries, review articles, original articles, case reports, the cutting edge: research update) Journal of Continuing Education in Health Professions (original research, reviews, innovations, forum, foundations, methodology, book reviews) Journal of Emergency Medicine (original research) Journal of Graduate Medical Education (brief reports, original research, ripout, innovation, review, on teaching and learning, perspective) Journal of the American Medical Association (original investigation, clinical trial, systematic reviews and meta-analysis, brief report) Medical Education (original research, review articles, cross-cutting edge, commentaries, letters) Medical Education Online (feature articles, research articles, trend articles, short communications, letters to the editor) Medical Science Educator (innovations, short communications, original research, monograph, commentary, letter to the editor, review) Medical Teacher (articles, short communications, letters to the editor, twelve tips, personal view, commentaries) Pediatric Emergency Care (original articles, illustrative cases, review articles) Scandinavian Journal of Trauma, Resuscitation and Emergency Medicine (commentary, review, letter to the editor, original research) Teaching and Learning in Medicine (groundwork, validation, investigations, educational case reports, observations) Western Journal of Emergency Medicine (original research, brief research report, case report, editorials (invited), educational advances, systematic reviews, letters to the editor)

**Table 2:** Outlets for digital dissemination.

Health Education Assets Library (HEAL): Digital library of multimedia teaching resources for the health sciences Journal of Education and Teaching in Emergency Medicine (JETem): Digital journal focused on medical education resources MedEdPORTAL: An open access educational resource for health care provider Multimedia Education Resource for Learning and Online Teaching (MERLOT II): Online repository and international consortium Portal of Geriatrics Online Education (POGOe): Elder care resource for interprofessional providers
